# Supplementary material for: Understanding links between water-quality variables and nitrate concentration in freshwater streams using high frequency sensor data
Source: PLoS One. 2023 Jun 30;18(6):e0287640. doi: 10.1371/journal.pone.0287640 (PMC10313027; doi:10.1371/journal.pone.0287640)
Supplement: S1 File — Time series of dissolved oxygen, nitrate concentration, specific conductance, surface water elevation, temperature, and turbidity in the three studied sites. (PDF) [file pone.0287640.s001.pdf]

Supporting Information for ”Understanding links between water-quality variables and nitrate concentration in freshwater streams using high-frequency sensor data”

SI 1 Original time series. **Time series of dissolved oxygen, nitrate concentration, specific conductance, surface water elevation, temperature, and turbidity in the three studied sites.**

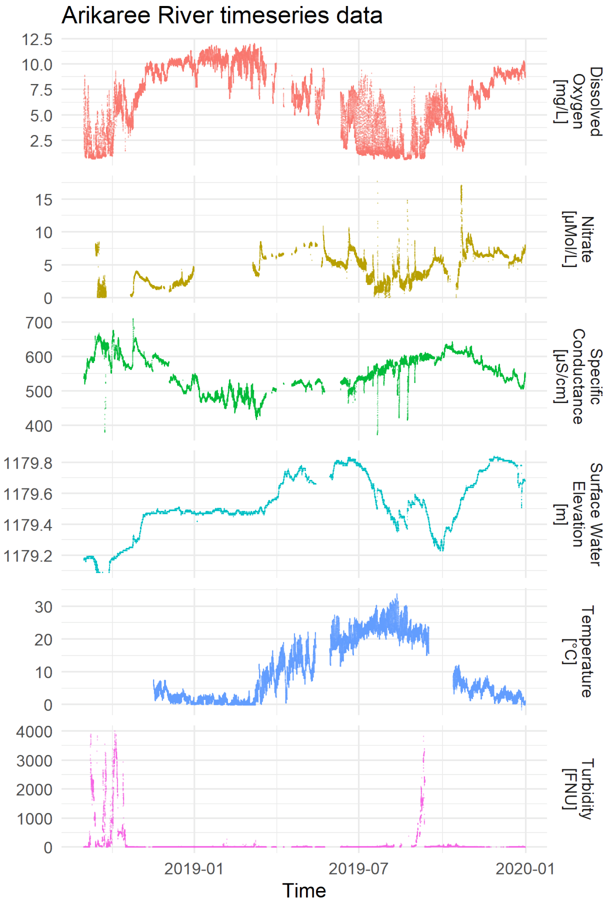
Arikaree River timeseries data
Dissolved Oxygen [mg/L]
Nitrate [µMol/L]
Specific Conductance [µS/cm]
Surface Water Elevation [m]
Temperature [°C]
Turbidity [FNU]
2019-01
2019-07
2020-01
Time

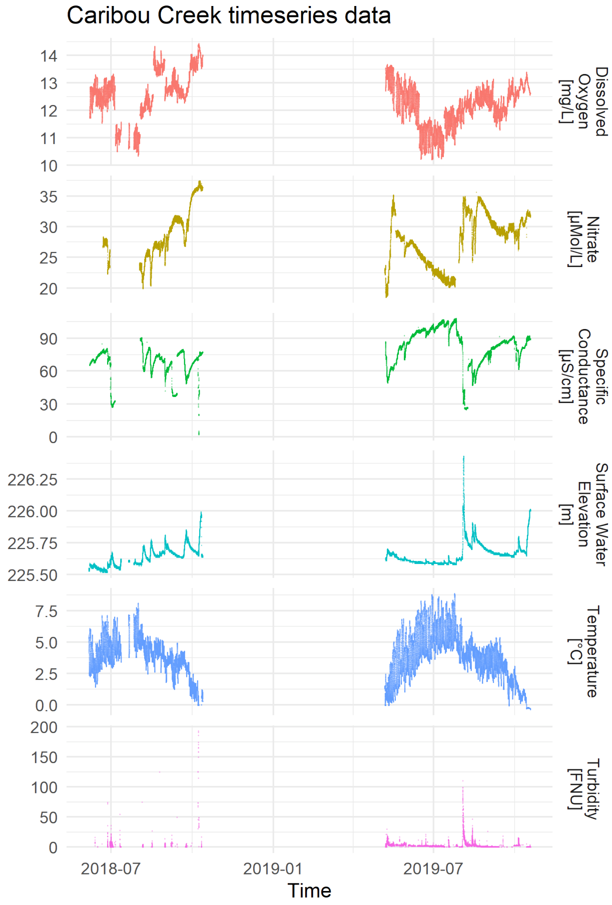
Caribou Creek timeseries data
Dissolved Oxygen [mg/L]
Nitrate [µMol/L]
Specific Conductance [µS/cm]
Surface Water Elevation [m]
Temperature [°C]
Turbidity [FNU]
2018-07
2019-01
2019-07
Time

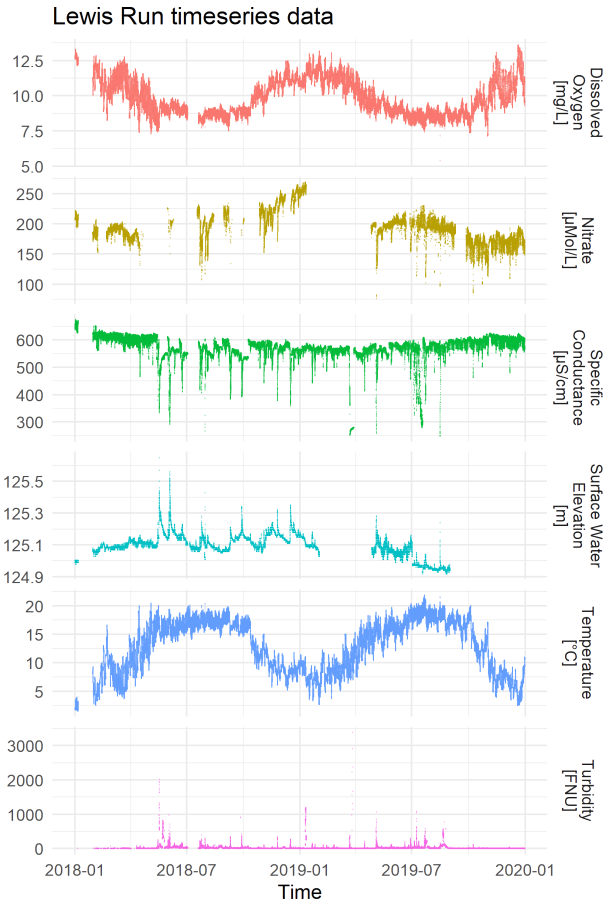
Lewis Run timeseries data
Dissolved Oxygen [mg/L]
Nitrate [µMol/L]
Specific Conductance [µS/cm]
Surface Water Elevation [m]
Temperature [°C]
Turbidity [FNU]
2018-01
2018-07
2019-01
2019-07
2020-01
Time
