# Supplementary material for: Understanding links between water-quality variables and nitrate concentration in freshwater streams using high frequency sensor data
Source: PLoS One. 2023 Jun 30;18(6):e0287640. doi: 10.1371/journal.pone.0287640 (PMC10313027; doi:10.1371/journal.pone.0287640)

Supporting Information for ”Understanding links between water-quality variables and nitrate concentration in freshwater streams using high-frequency sensor data”

SI 2 Diagnostic plots. Diagnostic plots of observed vs fitted values for the GAM and GAMM model constructed at each site.

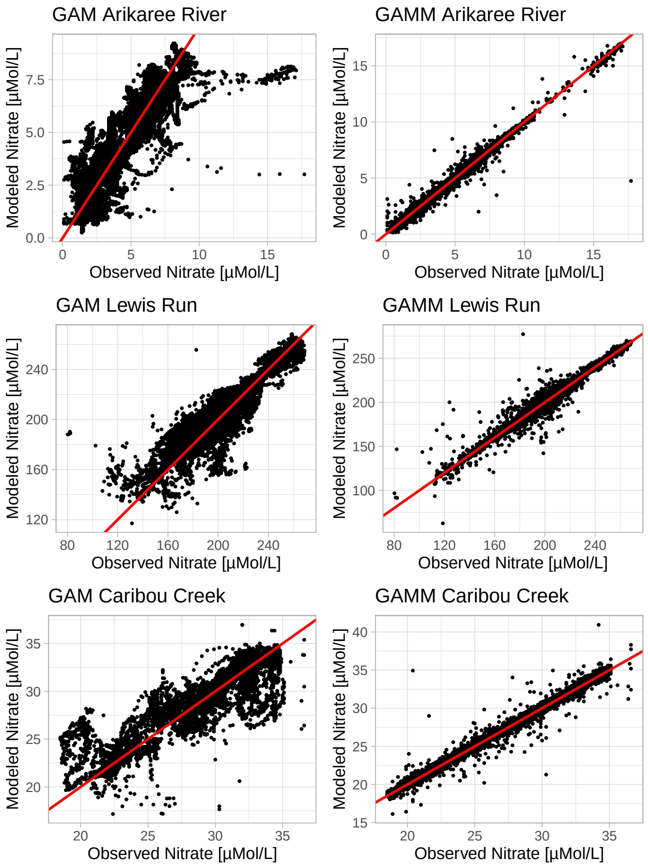

Supplement: S2 File — Diagnostic plots of observed vs fitted values for the GAM and GAMM model constructed at each site. (PDF) [file pone.0287640.s002.pdf]
